# Loss of Frizzled 9 in Lung Cells Alters Epithelial Phenotype and Promotes Premalignant Lesion Development

**DOI:** 10.3389/fonc.2022.815737

**Published:** 2022-07-18

**Authors:** Kayla Sompel, Lori D. Dwyer-Nield, Alex J. Smith, Alamelu P. Elango, Lauren A. Vanderlinden, Katrina Kopf, Robert L. Keith, Meredith A. Tennis

**Affiliations:** ^1^ School of Medicine, University of Colorado Anschutz Medical Campus, Aurora, CO, United States; ^2^ Skaggs School of Pharmacy, University of Colorado Anschutz Medical Campus, Aurora, CO, United States; ^3^ School of Public Health, University of Colorado Anschutz Medical Campus, Aurora, CO, United States; ^4^ Office of Academic Affairs, National Jewish Health, Denver, CO, United States; ^5^ Division of Pulmonary Sciences and Critical Care Medicine, Rocky Mountain Regional Medical Center, Aurora, CO, United States

**Keywords:** Frizzled 9, lung cancer, transformed growth, EMT, premalignant lesions

## Abstract

The transmembrane receptor Frizzled 9 (FZD9) is important for fetal neurologic and bone development through both canonical and non-canonical WNT/FZD signaling. In the adult lung, however, Fzd9 helps to maintain a normal epithelium by signaling through peroxisome proliferator activated receptor γ (PPARγ). The effect of FZD9 loss on normal lung epithelial cells and regulators of its expression in the lung are unknown. We knocked down FZD9 in human bronchial epithelial cell (HBEC) lines and found that downstream EMT targets and PPARγ activity are altered. We used a FZD9^-/-^ mouse in the urethane lung adenocarcinoma model and found FZD9^-/-^ adenomas had more proliferation, increased EMT signaling, decreased activation of PPARγ, increased expression of lung cancer associated genes, increased transformed growth, and increased potential for invasive behavior. We identified PPARγ as a transcriptional regulator of FZD9. We also demonstrated that extended cigarette smoke exposure in HBEC leads to decreased FZD9 expression, decreased activation of PPARγ, and increased transformed growth, and found that higher exposure to cigarette smoke in human lungs leads to decreased FZD9 expression. These results provide evidence for the role of FZD9 in lung epithelial maintenance and in smoking related malignant transformation. We identified the first transcriptional regulator of FZD9 in the lung and found FZD9 negative lesions are more dangerous. Loss of FZD9 creates a permissive environment for development of premalignant lung lesions, making it a potential target for intervention.

## Introduction

Frizzled 9 (FZD9) is a G protein-coupled transmembrane receptor commonly expressed in brain, testis, skeletal muscle and renal tissue (1). FZD9 is in the chromosomal region 7q11.23 and alterations are associated with Williams-Beuren syndrome, while loss of FZD9 in mouse models has been associated with slight abnormality in B-cell development, impaired osteoblast function, and learning defects (2). Alterations in FZD9 have been associated with cancers including astrocytoma, osteosarcoma, acute myeloid leukemia, and hepatocellular carcinoma (3–7). FZD9 interacts with several WNT ligands in tumors to activate β-catenin signaling, including WNT2, WNT5a, and WNT3a, and to promote EMT and invasiveness. In contrast, in the lung FZD9 interacts with WNT7a to activate tumor suppressive signaling. WNT7a binds to FZD9 and signals to peroxisome proliferator activated receptor gamma (PPARγ) through an Erk5-dependent cascade, leading to anti-tumor signaling, including increasing epithelial and reducing mesenchymal gene expression (3, 8). Loss of FZD9 in NSCLC cell lines leads to increased transformed growth and decreased PPARγ signaling, but these studies did not investigate the effect of FZD9 loss in a normal lung epithelium (9).

In matched human lung tumor and normal tissues, 77% of tumors had less FZD9 than normal tissues, suggesting that FZD9 is a lung tumor suppressor (10). In serial endobronchial biopsies, FZD9 expression is higher in regressive endobronchial dysplasia compared to progressive or persistent endobronchial dysplasia (10). Urethane and smoke exposed mice have decreased FZD9 expression (10). In an *in vitro* model using a human bronchial epithelial cell line (HBEC), FZD9 expression decreases with short and long term cigarette smoke condensate (CSC) exposure (10). Mutations in Frizzled receptors are not common in lung cancer and regulation of FZD9 in the lung is largely unknown. A few studies have identified regulators in other contexts, such as leukemia and bone formation (5, 11). To further characterize FZD9 in the lung and investigate its preventive and therapeutic potential, we studied the effects of FZD9 loss on normal lung cells and how it is affected by cigarette smoke using *in vitro* and *in vivo* models and human lung biopsies.

## Materials and methods

### Cell Culture

Non-transformed human bronchial epithelial cells (HBEC3KT and HBEC2KT) (a gift from the lab of Dr. John Minna, UT Southwestern) were cultured in Keratinocyte Serum Free Medium (GIBCO) (12). All HBEC cell cultures were grown and handled in a dedicated incubator. Human embryonic kidney 293T (HEK293T) cell line (purchased from the American Type Culture Collection) was cultured in DMEM (GIBCO) with 10% FBS. All cell lines were cultured at 37°C in a humidified 5% CO2 incubator and passaged twice per week. To generate cigarette smoke condensate (CSC), filters from a TE-10 smoking machine (Teague Enterprises) were weighed before and after smoking ten cigarettes and then soaked in DMSO to recover cigarette smoke particulate. For CSC exposure, 24 hours after seeding HBEC3KT, cells were treated with 5µg/mL of CSC or equal volume vehicle control (DMSO). Treatment was repeated after each passage, two times per week for one to 24 weeks and conducted in triplicate. Adenoma cell lines were created as previously described from WT and FZD9^-/-^ urethane treated mice (13). After they were established, adenoma lines were cultured in DMEM (GIBCO) with 10% FBS and 1% of 10,000U/mL of Pen/Strep (Gibco) and 1% of 25ug/mL of AmphotericinB (Gibco) at 37°C in a humidified 5% CO2 incubator and passaged twice per week. For the migration assay, wild type and FZD9^-/-^ adenoma cell lines were cultured in a 24 well plate with a 500μm silicone insert (Ibidi) and grown to 90% confluency around the insert. The insert was removed, and cells were imaged at 0, 4, 8, and 24 hours. Migration was captured on Nikon TMS microscope fitted with an AmScope digital camera. For the transformed growth assay, tumor cell lines were grown on a low-attachment plate (S-BIO) at a concentration of 1000 cells/well. At 72 hours, cell growth was analyzed using the CellTiter Glo Assay (Promega) (14). Statistical analysis was done by two-tailed t-test or one way ANOVA with Tukey’s *post hoc* analysis in GraphPad Prism (RRID : SCR_002798, version 9.0.2).

### qPCR

Mouse lung tissue and lesions were collected in RNA Later (Qiagen) at the time of harvest. Cell line and tissue RNA were extracted with the RNeasy Plus kit (Qiagen). qPCR Prime PCR Assays (Bio-Rad) for mouse included: NFKB, EZH2, FZD9, FN1, VIM, COX2, IL1β, ESR1, ESR2, SNAI1, PLK1, Cyclin D1, BCL2, NCAD, ECAD, VEGFA, COL1α2, and PPARγ. qPCR was conducted using standard protocol for SsoAdvanced SYBR Green Master Mix (Bio-Rad) on a CFX96 Touch (Bio-Rad). All gene expression data was normalized to the reference gene RPS18 and fold changes were calculated using the 2^-ΔΔCt^ method. PCR analysis was conducted in triplicate and statistical analysis was done by two-tailed t-test or one way ANOVA with Tukey’s *post hoc* analysis in GraphPad Prism.

### Western Blot, ELISA, Dot Blot

Mouse lung tissue was collected and flash frozen in liquid nitrogen at the time of harvest. Protein was extracted from 10mg of tissue and for western blots, 20ug per sample was used to measure vimentin, COX2, e-cadherin, and β-actin (vimentin, 1:1000 Cell Signaling Technologies#D21H3; COX2, 1:500 Protein Tech#6635-1-Ig; e-cadherin, 1:3000 Protein Tech#20874-1-AP; β-actin, Biorad #MCA5775GA). Quantitative analysis was performed on triplicate experiments by creating a ratio between the band intensity for the protein of interest and its corresponding β-actin band intensity. Additional protocol details for western blots are included in the supplementary methods. For the active β-catenin ELISA, protein was extracted from 10mg of flash frozen mouse tissue and 80% confluent 100mm plates of tumor cells using Symansis MKA buffer (Symansis). After protein quantification by BCA assay, the ELISA was performed with 100μg of mouse tissue protein or 30μg of tissue lysate per manufacturer instructions (Symansis). Fluorescence was measured on a Glomax Instrument (Promega). For the apoptosis protein dot blot, protein was extracted from tumor cells using RIPA buffer (Thermo Scientific) with HALT protease inhibitor (Thermo Scientific). After protein quantification by BCA assay, one apoptosis dot blot assay was performed for each sample per the manufacturer’s instructions (RayBiotech, #AAM-APO-1-8) with 500μg of cell lysate. Blots were imaged with chemiluminescence settings on a BioRad ChemiDoc Imager. Relative differences in protein were determined by normalizing the positive control intensity between membranes.

### Transfections

For PPRE assays, HBEC3KT and HBEC2KT cells were seeded at 5000 cells/well in a 96 well plate with triplicates for each experimental group. After 24 hours, wells were transfected with PPARγ response element luciferase (PPRE; a gift from Bruce Spiegelman; Addgene plasmid #1015) (45ng) and renilla control reporter vector (Promega) (5ng) using TransIT-X2 transfection reagent (Mirus Bio) per the manufacturer’s protocol. After 48 hours, PPRE (firefly) and control reporter (renilla) were measured using the Dual-Luciferase Reporter assay kit (Promega) on a Glomax instrument (Promega). For CSC experiments in HBEC3KT, cells were treated with 5μg/ml of CSC for 48 hours or two weeks before luciferase analysis. For FZD9 promoter experiments, HBEC3KT were transfected with a luciferase vector containing the complete Fzd9 promoter, PPARγ plasmid (Genecopoeia), or control plasmid (Genecopoeia), with activity measured by the Secret-Pair Dual Luminescence Assay kit (Genecopoeia) on a Glomax. siRNA experiments were transfected with FZD9 siRNA (Genecopoeia), PPARγ siRNA (Qiagen), or Allstar negative siRNA control (Qiagen). Experiments were conducted in triplicate. Significance of luciferase assays was assessed by two-tailed t-test or one way ANOVA with Tukey’s *post hoc* analysis in GraphPad Prism.

### *In Vivo* Mouse Studies

The methods for generation and genotyping of FZD9^-/-^ FVBN mice are in the supplementary methods. Male and female FZD9^-/-^ mice were generated for experiments by breeding FZD9^-/-^ males with FZD9^-/-^females to produce 100% knockout litters and were housed in a pathogen-free facility in the Veterinary Care Unit at the Rocky Mountain Regional VA Medical Center (RMRVAMC). Mice were injected IP with 100μl of 1 mg urethane/g body weight dissolved in 0.9% saline vehicle or with 100ul saline. Mice were weighed daily for 7 days after urethane injection and weekly for the remainder of the experiment. After 16 weeks of urethane exposure, mice were sacrificed by a lethal dose of Fatal Plus. Lungs from three mice were inflated with formalin and fixed. Lesions were dissected from surrounding lung tissue and diameters were measured with digital calipers. The surrounding lung tissue was saved for RNA extraction. For the six-week smoke study, one group of mice was exposed to whole body cigarette smoke (CS) at particulate levels of 35 mg/m^3^ and the other to ambient air in Teague Enterprises TE-10 smoking machines for 6 hour/day, 5 days/week for 6 weeks. Mice were weighed daily during the first 3 weeks of CS exposure, and weekly thereafter. CS exposure was suspended in mice experiencing 15-20% weight loss, until they regained weight. RNA was extracted from whole lung tissues. Statistical analysis using a two-tailed t-test or one way ANOVA with Tukey’s *post hoc* analysis was conducted using GraphPad Prism. Studies were carried out in accordance with the recommendations in the NIH Guide for the Care and Use of Laboratory Animals and were approved by the RMRVAMC Animal Care and Use Committee. microCT imaging is described in the supplementary methods. Wild type and FZD9^-/-^ tumor cell lines were generated by dissecting 24-week urethane induced tumors, chopping tumors into 1mm sections, and culturing in DMEM, low glucose, 10% FBS media until cells grew consistently in a 100mm dish and had 20+ passages.

### Immunohistochemistry

5μm lung sections were used for Ki67 staining (1:2000 dilution, Abcam 15580). Tumor area was measured and Ki67-positive nuclei/mm^2^ were counted in each tumor. Replicate blinded counts were conducted. The Ki67+ nuclei/mm^2^ tumor area for each tumor was averaged by group. A one-way ANOVA with Tukey’s *post hoc* analysis in GraphPad Prism was used to determine statistical differences between groups. H&E stains were done on 5μm lung sections from WT and FZD9^-/-^, urethane and saline groups. Additional protocol details are included in the supplementary methods.

### Serum PPARγ Activity Assay

Serum was collected by intracardiac puncture immediately after euthanizing each mouse. HEK293T cells were seeded at 2,000 cells/well in a 96 well plate. After twenty-four hours, the cells were transfected with 45ng of PPRE and 5ng renilla control reporter vector using TransiT-X2 transfection reagent and Opti-MEM media, per the manufacturer’s protocol. Cells were treated with 10μl mouse serum at 24 hours and 48 hours post transfection (15). Serum treatments were conducted in triplicate and included empty and mock transfection controls. Luciferase activity was measured after 48 hours using the Dual-Luciferase Reporter assay kit on the Glomax. PPRE activity was normalized to renilla activity and analyzed relative to untreated controls. Significance was assessed by two tailed t-test in GraphPad Prism.

### Analysis of Human Lung Biopsy Samples

The oral iloprost lung cancer chemoprevention study was a Phase II, placebo-controlled trial of iloprost in current and former smokers at increased risk for lung cancer (NCT00084409). The characteristics of the population, protocol, and the results have been reported in detail (16). Biopsies were collected at baseline and after six months of treatment with iloprost or placebo. Pre- and post-treatment biopsies were collected for 125 subjects and 413 total tissues were available for analysis. RNA *in situ* hybridization (RISH) was conducted to measure FZD9 and PPARγ in biopsy tissues, which were deidentified and used with approval of the Colorado Multiple Institutional Review Board. RNAscope GAPDH (positive control), dapB (negative control), FZD9, and PPARγ probes were used according to the manufacturer’s protocol (ACDBio). Percent of epithelial cells with signal in each sample were counted and samples were included in analysis if the positive control and targets had at least 10% more positive cells than the negative control. RISH Data Pre-processing and Quality Control: Quantitation of gene expression was used by summing up the number of slides with the gene present. GAPDH was a reference gene and if the negative control was more than 5 units above GAPDH (n=14) or the expression for GAPDH was missing (n=25), the samples was removed from the dataset. Further QC was done on the individual gene level with any samples missing removed from analysis (FZD9 n = 14, PPARγ n = 24). Gene expression, after adjustment for GAPDH, was used moving forward for all statistical modeling.

### Baseline Statistical Model

In our baseline model, gene expression was dichotomized into low or high gene expression based on the median value. Pack years and quit time were dichotomized into either high or low based on the median value for each variable. Each smoking variable of interest (smoking status, pack years and quit time), a logistic mixed-model was run (lme4, v.1.1-26) using gene expression as the outcome and smoking variable as the predictor while adjusting for sex and accounting for random effects of subject. For quit time, we flipped the OR for comparison purposes with the other smoking variables (i.e., high smoking exposure is the risk factor modeled).

## Results

### Loss of FZD9 in Normal Lung Epithelial Cells Alters EMT and PPARγ Pathways

**Figure 1:**
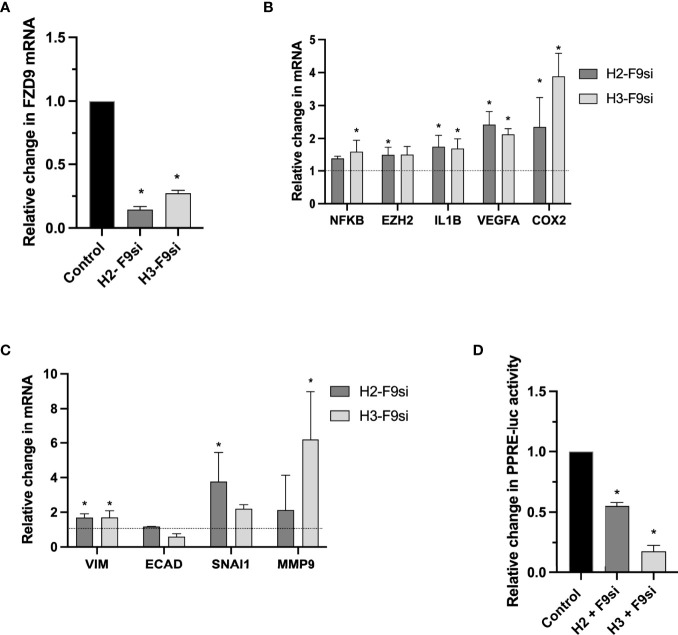
Loss of FZD9 alters downstream targets in HBEC. siRNA was used to knock down FZD9 expression in HBEC3KT and HBEC2KT cell lines and results are shown relative to a scramble siRNA control. **(A)** FZD9 expression measured by qPCR. Results are normalized to GAPDH. **(B)** Gene expression with FZD9 siRNA transfection was measured for NFKB, EZH2, IL1B, VEGFA and COX2. Results are normalized to GAPDH and relative to a scramble siRNA control (dotted line). **(C)** Expression of VIM, ECAD, SNAI1 and MMP9 was measured after FZD9 siRNA transfection. Results are normalized to GAPDH and relative to a scramble siRNA control (dotted line). **(D)** Change in PPRE-luciferase activity. Each cell line is shown relative to its own control transfection (set to 1). Transfections and qPCR were conducted in triplicate. H2-F9si, HBEC2KT + FZD9 siRNA; H3-F9si, HBEC3KT + FZD9 siRNA. Statistical significance was measured by a two tailed t-test relative to each cell line’s control. Significance is *p<0.05 compared to control.

Loss of FZD9 in NSCLC cell lines leads to decreased PPARγ activity, but the effect of FZD9 loss on non-transformed cells is unknown (9). We knocked down expression of FZD9 in two human bronchial epithelial cell lines (HBEC3KT and HBEC2KT) using an siRNA approach (**Figure 1A**). HBEC cells are immortalized by co-transfecting with cyclin-dependent kinase 4 (CDK4) and human telomerase reverse transcriptase (hTERT). This allows HBEC cells to replicate continuously with epithelial-like features but without becoming malignant (17). Loss of FZD9 in these cell lines altered expression of lung cancer associated genes and targets of PPARγ, a downstream measure of FZD9 activity (**Figure 1B**). In both cell lines, loss of FZD9 led to significant increases in IL1β, VEGFA, and COX2 and in the HBEC3KT cell line, NFKβ and EZH2 also had significantly increased expression. Loss of FZD9 led to alterations in EMT gene expression in both cell lines (**Figure 1C**). Mesenchymal gene vimentin (VIM) increased in both cell lines, while snail increased in HBEC2KT and MMP9 increased in HBEC3KT. Epithelial gene e-cadherin (ECAD) decreased slightly with FZD9 loss in HBEC3KT. Gene expression changes varied between the two cell lines but shared some similarities and both demonstrated effects of FZD9 loss. Loss of FZD9 in both cell lines decreased the activity of PPARγ as measured by PPRE (**Figure 1D**). These results demonstrate that loss of FZD9 in lung epithelial cells leads to changes associated with lung cancer and could support EMT. FZD9 loss in combination with a carcinogen exposure could augment tumor promotion.

### Loss of FZD9 *In Vivo* Increases Adenoma Multiplicity

**Figure 2:**
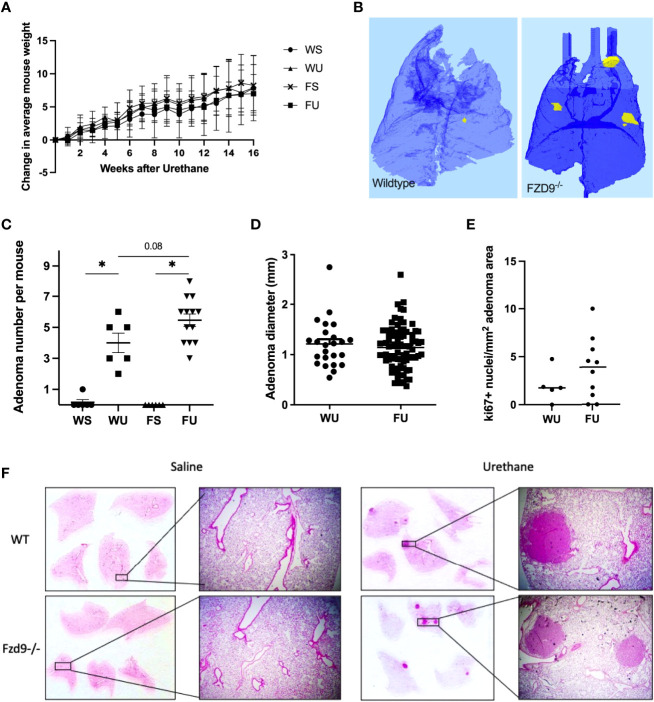
Loss of FZD9 increases adenoma multiplicity *in vivo*. **(A)** Mice were weighed weekly after urethane exposure and average change each week was calculated for each group. **(B)** Representative microCT image of wild type and FZD9^-/-^ mouse lungs 16 weeks after urethane exposure. Yellow indicates presence of an adenoma. **(C)** Wild type and FZD9^-/-^ mice were exposed to 16 weeks of urethane or saline control and lesions were counted after sacrifice. Statistical significance was measured by one way ANOVA with Tukey’s *post hoc* analysis and *p<0.05. **(D)** The diameter of each lesion was measured and compared between wild type and FZD9^-/-^ mice. **(E)** Positive Ki-67 nuclei per mm^2^ lesion area from adenomas in wild type or FZD9^-/-^ mice exposed to urethane. **(F)** Representative H&E stains of whole lungs and 10x magnification from each group. WS, wild type saline; WU, wild type urethane; FS, FZD9^-/-^ saline; FU, FZD9^-/-^ urethane.

To validate *in vitro* effects of FZD9 loss in normal lung cells, we used a CRISPR approach to generate an FVB/N FZD9^-/-^ mouse. Loss of FZD9 had no significant physiologic or anatomic effects up to one year of age. FZD9^-/-^ mice, along with wild type controls, were used in an established lung adenocarcinoma model, where animals are given a single injection of urethane or saline control and develop adenomas starting at 6 weeks, with carcinomas developing at 20 weeks (18). There were no differences in animal weights during the experiment (**Figure 2A**). **Figure 2B** shows a 3D image generated from microCT imaging that is representative of adenoma development in wild type and FZD9^-/-^ mice treated with urethane. Urethane exposure in wild type or FZD9^-/-^ mice leads to increased adenoma multiplicity compared to saline controls (**Figure 2C**). Adenoma multiplicity in FZD9^-/-^ urethane mice compared to wild type urethane mice trended higher (p=0.08) (**Figure 2C**). Adenoma diameter did not differ between urethane exposed wild type or FZD9^-/-^ mice (**Figure 2D**). Ki67 proliferation staining in adenomas was not significantly different between urethane exposed wild type and FZD9^-/-^ mice (**Figure 2E**). FZD9^-/-^ adenomas displayed a wide range of Ki67 staining, suggesting there may be subsets of FZD9^-/-^ lesions with other alterations that stimulate proliferation (Representative Ki-67 images, **Supplementary Figure 1**). **Figure 2F** shows representative H&E stains of whole lung and 10x magnification from wild type saline, FZD9^-/-^ saline, wild type urethane, and FZD9^-/-^ urethane animals.

### Downstream Gene Expression and Activity Is Affected by FZD9 Loss *In Vivo*

**Figure 3:**
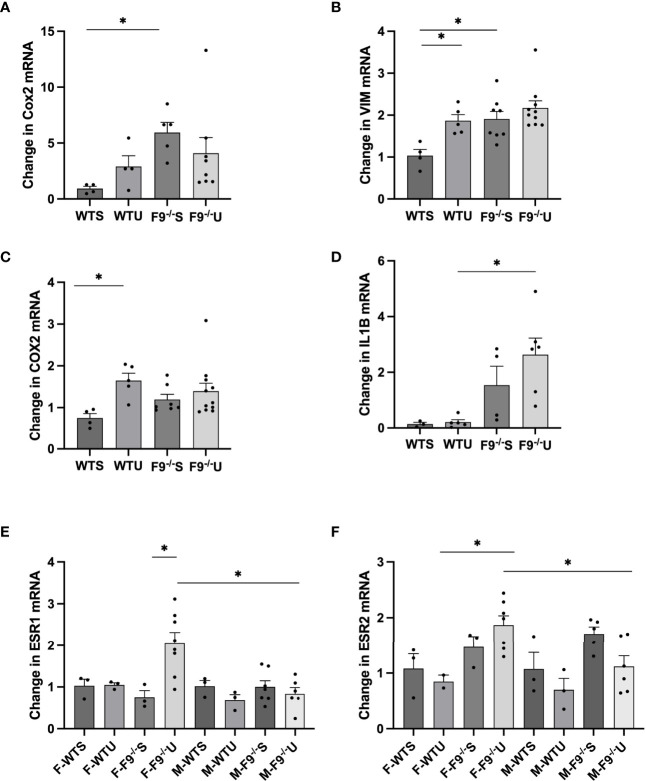
FZD9 loss *in vivo* alters mRNA expression of downstream targets. RNA from mouse lung was harvested from wild type saline (WTS), wild type urethane (WTU), FZD9^-/-^ saline (F9^-/-^S), and FZD9^-/-^ urethane (F9^-/-^U) groups and gene expression measured by qPCR. **(A)** FN1, **(B)** VIM, **(C)** COX2, **(D)** IL1B, **(E)** ESR1, **(F)** ESR2. PCR was normalized to RPS18 and measured in triplicate. F-, Female; M-, Male. Significance was measured by one-way ANOVA and *p<0.05.

FZD9 activates PPARγ, which is associated with reducing inflammatory signals and reversing EMT signals. We measured gene expression markers of EMT and inflammation in whole lung samples from wild type saline, FZD9^-/-^ saline, wild type urethane, and FZD9^-/-^ urethane mice. We found that mesenchymal marker fibronectin (FN1) increased significantly with loss of FZD9 compared to wild type mice (**Figure 3A**). FN1 also increased, though non significantly, with exposure to urethane in wild type mice and was slightly higher in FZD9^-/-^ mice with urethane. Mesenchymal marker vimentin (VIM) significantly increased in wild type urethane mice compared to wild type saline mice and was elevated in FZD9^-/-^ saline mice similarly to wild type urethane exposure (**Figure 3B**). Inflammatory marker COX2 was significantly elevated with urethane in wild type mice and trended toward increased levels in FZD9^-/-^ mice with saline or urethane (**Figure 3C**). Inflammatory marker IL1β was non-significantly elevated with urethane in wild type mice and with FZD9 loss, but was significantly higher in FZD9^-/-^ mice exposed to urethane compared to wild type mice exposed to urethane (**Figure 3D**). Previous gene expression screening suggested estrogen receptor expression may be increased by FZD9 loss and alterations in estrogen receptors have been associated with lung cancer, so we measured expression of estrogen receptor α (ESR1) and β (ESR2) (19). We found no difference in ESR1 expression in female or male wild type mice exposed to urethane but detected significantly elevated expression of ESR1 in female FZD9^-/-^ mice exposed to urethane compared to female Fzd9^-/-^ saline mice and to male FZD9^-/-^ urethane exposed mice (**Figure 3E**). Expression of ESR2 was significantly elevated in female FZD9^-/-^ urethane mice compared to wild type urethane mice and compared to male Fzd9^-/-^ urethane mice (**Figure 3F**). In male mice, expression of ESR2 trended toward an increase in FZD9^-/-^ saline mice compared to wild type saline mice and toward a decrease with urethane exposure in both genotypes (**Figure 3F**).

**Figure 4:**
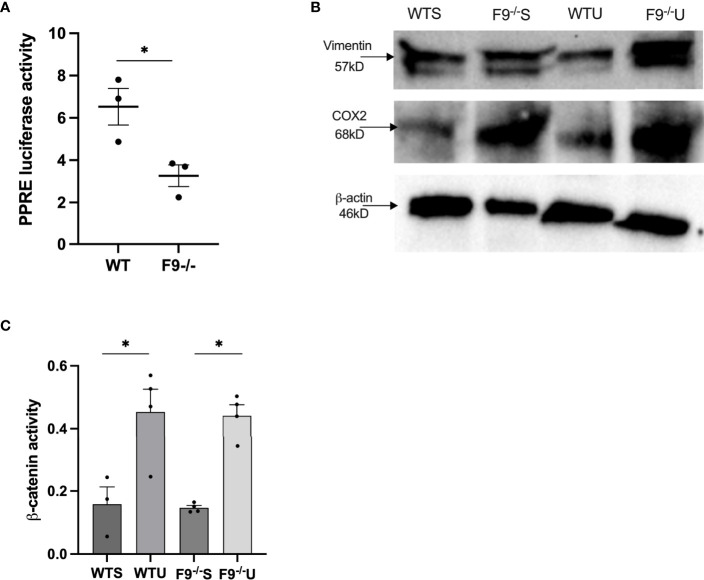
Loss of FZD9 *in vivo* alters downstream target activity. **(A)** HEK293t cells were transfected with PPRE-luc and treated with 10ul mouse serum from wild type or FZD9^-/-^ mice. Significance was measured by two-tailed t-test and *p<0.05 **(B)** Protein was extracted from whole lung samples from wild type or FZD9^-/-^ mice exposed to saline or urethane and analyzed for vimentin and COX2 protein expression, with β-actin as a loading control. **(C)** Active β-catenin was measured by ELISA in wild type or FZD9^-/-^ mice treated with saline or urethane. The assay was conducted in triplicate. Significance was measured by one-way ANOVA with Tukey’s *post hoc* analysis and *p>0.05. WT, wild type; F9-/-, FZD9 knockout; WTS, wild type saline; F9^-/-^S, FZD9^-/-^ saline; WTU, Wild type urethane; F9^-/-^U, FZD9^-/-^ urethane.

To measure effects of FZD9 loss on downstream PPARγ activity, serum was collected from wild type and FZD9^-/-^ mice and used to treat HEK293t cells transfected with PPRE (15). PPRE activity resulting from treatment with serum from FZD9^-/-^ was significantly lower than wild type mice, indicating that there is a lower level of PPARγ activation when FZD9 is lost *in vivo* (**Figure 4A**). To further explore the downstream effects of FZD9 loss *in vivo*, we measured protein levels of mesenchymal marker VIM and inflammatory marker COX2 in whole lung protein extracts from wild type saline, FZD9^-/-^ saline, wild type urethane, and FZD9^-/-^ urethane mice. Loss of FZD9 increased COX2 expression with urethane exposure when compared to saline (**Figure 4B**).

VIM expression increased with urethane exposure in wild type mice and was higher in FZD9^-/-^ mice (**Figure 4B**). FZD9 in the lung epithelium does not activate canonical β-catenin signaling, but pro-tumorigenic β-catenin activation may be a contributor to the tumor supportive conditions that accompany FZD9 loss (3, 8, 9, 20). In **Figure 4C**, we show increased active β-catenin in whole lung with exposure to urethane in both wild type and FZD9^-/-^ mice. There was no difference in active β-catenin with FZD9 loss in saline or urethane mice compared to wild type. Together, these data suggest the environment in FZD9^-/-^ lungs has altered gene expression and downstream activity that may support development of lung adenomas, but that loss of Fzd9 expression alone does not increase β-catenin activity.

### FZD9^-/-^ Adenomas Have More Characteristics of Tumors

**Figure 5:**
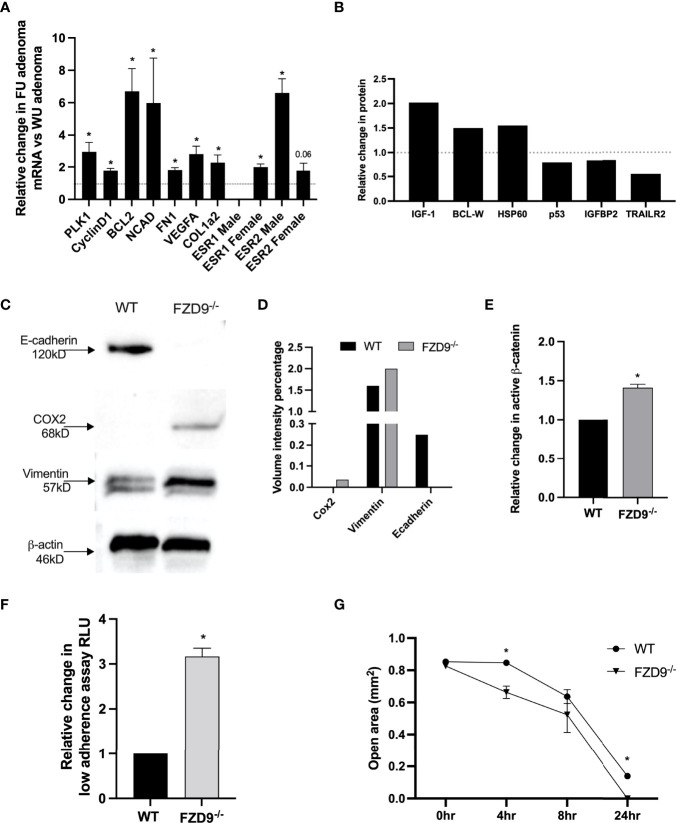
Urethane induced FZD9^-/-^ adenomas have a more tumor-like phenotype. **(A)** Change in gene expression in FZD9^-/-^ (FU) adenomas relative to wild type (WU) adenomas, measured by qPCR. Data is normalized to RPS18 and assays were conducted in triplicate. **(B)** Changes in apoptosis pathway proteins was measured by dot blot, normalized to dot blot positive controls, and quantified. (n=1) **(C)** Change in protein expression measured by western blot of FZD9^-/-^ and WT adenoma cell lines. β**-**actin is a loading control. **(D)** Quantification of bands from western blot in **(C)**. **(E)** Active β-catenin was measured by ELISA in a Fzd9^-/-^ adenoma cell line and is shown relative to a WT adenoma cell line. The assay was conducted in triplicate. **(F)** Growth on low adherence plates measured by fluorescence viability assay in a FZD9^-/-^ adenoma cell line and shown relative to a WT adenoma cell line. Assay was conducted in triplicate. **(G)** Migration was measured in triplicate by quantifying the open area left by a silicone insert in cells cultured over 24 hours. WT, wild type tumor cell line; F9^-/-^, FZD9 knockout tumor cell line. Statistical significance was measured by a two-tailed t-test and *p<0.05.

In the urethane lung cancer mouse model at 16 weeks after injection, FVB/N mice have multiple hyperplasias and adenomas, while saline controls typically have none. We collected adenomas from wild type urethane and FZD9^-/-^ urethane mice, extracted RNA, and used qPCR to measure expression of lung cancer associated genes. FZD9^-/-^ adenomas had significantly higher expression of polo-like kinase 1 (PLK1) and cyclin D1, which are associated with altered cell cycle control in lung cancer (**Figure 5A**). Increased PLK1 is also associated with persistent squamous dysplasias (21). BCL2 is elevated in FZD9^-/-^ adenomas, suggesting increased avoidance of apoptosis (**Figure 5A**). Significantly elevated N-cad, FN1, and VEGF may contribute to increased potential of FZD9^-/-^ adenomas for progression to carcinoma (**Figure 5A**). Increased COL1a2 may result from the presence of cancer associated fibroblasts in FZD9^-/-^ adenomas and is associated with poor prognosis (22). ESR1 is overexpressed in NSCLC and promotes proliferation, migration, and invasion of lung cancer cells (23). ESR1 was not detected in male FZD9^-/-^ adenomas and was significantly elevated in female FZD9^-/-^ adenomas (**Figure 5A**). ESR2 expression is higher in normal lung, is associated with poor prognosis of NSCLC, and is elevated in male adenocarcinomas (19). Here, ESR2 was elevated significantly in male FZD9^-/-^ adenomas and non-significantly in female FZD9^-/-^ adenomas (**Figure 5A**). We generated cell lines from adenomas collected from wild type and FZD9^-/-^ mice. These cell lines were compared for differences in protein expression of downstream targets and for changes in cell behavior. Increased BCL2 in FZD9-/- adenomas suggested an effect of FZD9 loss on cell survival, so we measured apoptosis proteins in the WT and FZD9^-/-^ adenoma cell lines by protein dot blot. In the FZD9^-/-^ adenoma cell lines compared to the WT cell line, we found increased IGF-1, BCL-W and HSP60, and decreased p53, IGFBP2, and TRAILR2, all supporting that loss of FZD9 contributes to increased cell survival in adenomas (**Figure 5B**) (Dot blot images are in **Supplementary Figure 2**). Western blot protein analysis of the adenoma cell lines indicated higher expression of e-cadherin in wild type adenoma cells and higher expression of COX2 and VIM in FZD9^-/-^ adenoma cells (**Figures 5C, D**). An active β-catenin ELISA detected increased active β-catenin in the FZD9^-/-^ adenoma cells compared to the wild type adenoma cells (**Figure 5E**). Adenoma cell lines were cultured for 48 hours on a low-adherence plate to measure transformed growth. FZD9^-/-^ adenoma cells had significantly higher growth compared to wild type adenoma cells (**Figure 5F**). To measure the migration capacity of the adenoma cell lines, a silicone insert was used to generate identical spaces while cells grew to 90% confluency around the insert. Over 24 hours, FZD9^-/-^ adenoma cells moved into an empty space on a tissue culture plate faster than wild type adenoma cells (**Figure 5G**) (Representative images of the migration assay are in **Supplementary Figure 3**). Together, these data suggest that FZD9^-/-^ adenomas have increased capacity for aggressive behavior and have higher levels of factors that promote tumor progression.

### CSC Decreases FZD9 Expression and Downstream Activity

**Figure 6:**
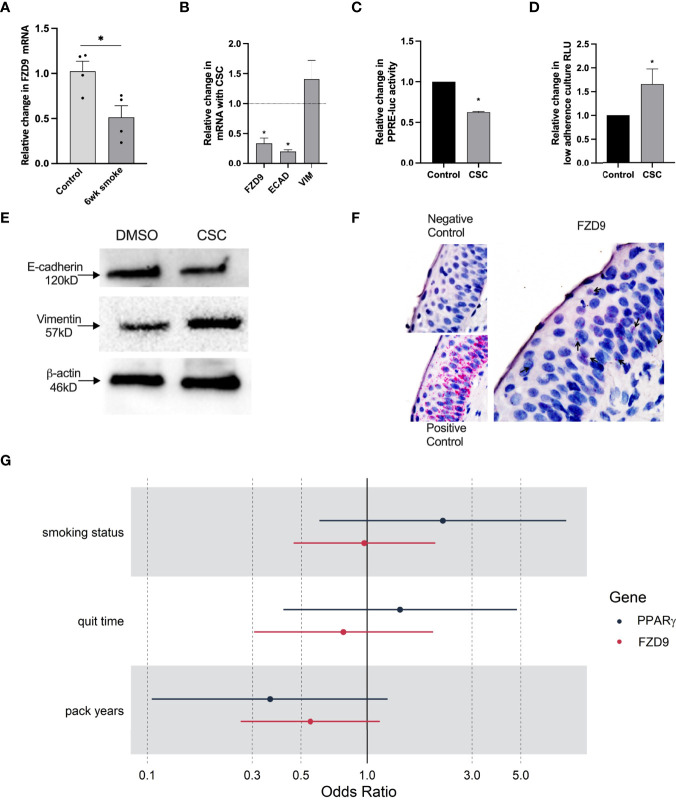
FZD9 expression decreases with smoke exposure. **(A)** Change in FZD9 expression in mice after six weeks of exposure to cigarette smoke or ambient air control, measured by qPCR. Results are normalized to RPS18 and conducted in triplicate. **(B–D)** HBEC3KT were treated with 5ug/ml CSC or DMSO every 48 hours for two weeks and analyzed for **(B)** FZD9, Ecad, and VIM expression by qPCR, **(C)** PPRE-luc activity by transfection, **(D)** viability in a low-adherence culture, and **(E)** Ecad and VIM protein expression by western blot, with β-actin loading control. Significance in A-E was measured by a two-tailed t-test and *p<0.05. CSC, cigarette smoke condensate. Treatments, transfections, luciferase assays, and qPCR was conducted in triplicate. qPCR results are normalized to GAPDH. Data is shown relative to control. **(F)** Representative images for RISH signal in negative control, positive control, and FZD9. **(G)** Odds ratios for the effect of smoking characteristics on FZD9 and PPARγ expression at baseline, where someone with higher pack year exposure to smoke has lower odds of high FZD9 expression.

*In vivo*, exposure to cigarette smoke carcinogens, including urethane and one week of smoke exposure, decreased FZD9 expression (10). Here, we measured FZD9 expression in FVB/N mice after six weeks of cigarette smoke exposure and found decreased FZD9 expression compared to mice exposed only to ambient air (**Figure 6A**). Exposure to CSC alters expression of FZD9 in HBEC3KT after 24 weeks of exposure (10). We investigated the effects of two weeks of CSC exposure on HBEC3KT. Expression of FZD9 and ECAD decreased significantly, while VIM expression increased (**Figure 6B**). We also demonstrated that two weeks of CSC exposure in HBEC3KT led to decreased PPARγ activity and increased growth in low adherence conditions (**Figures 6C, D**). This was also validated with protein extraction, where ECAD was decreased and VIM was increased after the cells were exposed to CSC (**Figure 6E**).

To understand effects of cigarette smoke on FZD9 and PPARγ expression in human lung epithelium, we used RNA *in situ* hybridization (RISH) to measure expression in lung biopsies from the oral iloprost clinical trial (16). We conducted RISH for GAPDH (positive control), dapB (negative control), PPARγ, and FZD9 on baseline and follow up biopsies from 125 patients (Representative staining, **Figure 6F**). We compared FZD9 and PPARγ expression in epithelial cells at baseline with smoking parameters, including smoking status (current or former), smoking pack years, and smoking quit time, to determine if there is an effect of cigarette smoke exposure on FZD9 or PPARγ expression. All analyses were adjusted for sex. Baseline model analysis of FZD9 produced non-significant results, however, we see a trend in all models where higher smoke exposure leads to lower FZD9 expression at baseline (**Figure 6G**). The estimated odds ratios (OR) for current smoking status and lower quit time were 0.97 (p=0.933) and 0.78 (p=0.577), respectively. The OR for pack years approached significance at 0.55 (p=0.086). PPARγ baseline models also resulted in non-significant results, where current smoking status and lower quit time led to higher expression of PPARγ (**Figure 6G**). The ORs for current smoking status and lower quick time were 2.21 (p=0.192) and 1.41 (p=0.582)), respectively. In contrast, higher pack years resulted in a lower expression of PPARγ that neared significance with a OR of 0.36 (p=0.08), which agrees with the FZD9 baseline model analysis. Analysis of the effect of cigarette smoke on the lung epithelium in human cells and tissue suggests that FZD9 expression is a target of cigarette smoke induced carcinogenesis.

### CSC Alters Expression of FZD9 Through PPARγ

**Figure 7:**
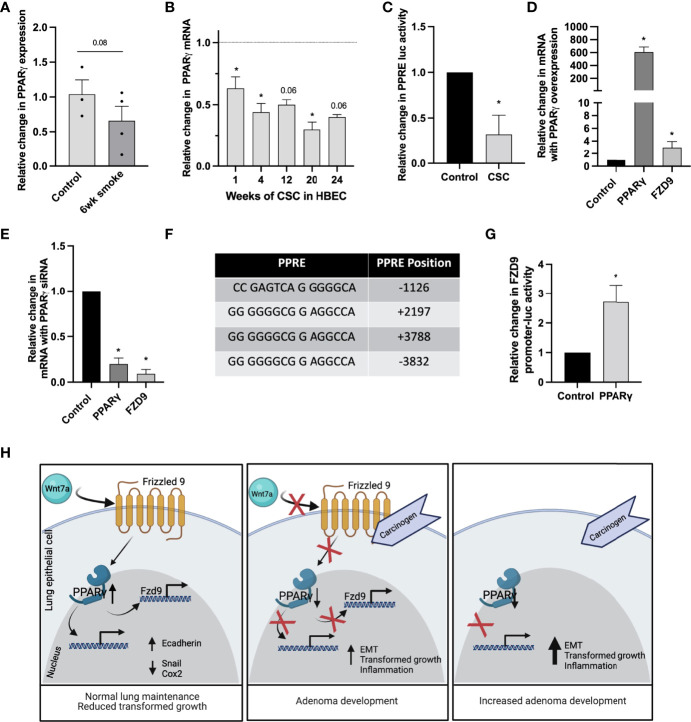
Transcription of FZD9 is decreased by CSC and increased by PPARγ. **(A)** Change in PPARγ expression in mice after six weeks of exposure to cigarette smoke or ambient air control measured by qPCR. Results are normalized to RPS18 and measured in triplicate. **(B)** Change in PPARγ expression in HBEC3KT with 1-24 weeks exposure to CSC relative to DMSO control at each time point (dotted line), measured by qPCR. Results are normalized to GAPDH and conducted in triplicate. **(C)** Change in PPRE-luc activity after 48 hours of CSC exposure relative to DMSO control in HBEC3KT. Transfections and luciferase assays were conducted in triplicate. **(D, E)** Fold change in PPARγ and FZD9 expression in HBEC3KT measured by qPCR after transfection in triplicate with **(D)** PPARγ or **(E)** PPARγ siRNA. Results are normalized to GAPDH, relative to control, and conducted in triplicate. **(F)** Putative PPREs in the TSS-flanking region of FZD9. **(G)** FZD9 promoter luciferase activity vs control in HBEC3KT after transfection with a PPARγ plasmid. **(H)** Diagram of the effects of carcinogen exposure and Fzd9 loss in lung epithelial cells. Diagram created with Biorender.com. Transfections and luciferase assays were conducted in triplicate. Statistical significance was determined by a two-tailed t-test or one-way ANOVA with Tukey’s *post hoc* analysis and *p<0.05. CSC, cigarette smoke condensate.

PPARγ activation in the lung epithelium occurs when Wnt7a binds FZD9, but if FZD9 expression is decreased by CSC, we hypothesized that PPARγ activity would also be lost. Loss of PPARγ occurs *in vitro* in lung epithelial cells with 16 weeks of CSC exposure and *in vivo* with urethane exposure (24). Here, we show decreased PPARγ expression with six weeks of cigarette exposure in mice (**Figure 7A**) and decreased expression in HBEC3KT with 1-24 weeks of CSC exposure (**Figure 7B**). We also found that 48 hours of CSC exposure in HBEC3KT significantly reduces PPRE activity compared to control cells (**Figure 7C**). Regulation of FZD9 transcription in the lung epithelium by transcription factors has not been described. We transfected HBEC3KT with a PPARγ expression plasmid, confirmed increased PPARγ expression, and found that FZD9 expression also increased (**Figure 7D**). To determine if PPARγ is necessary for FZD9 expression, we transfected a PPARγ siRNA into HBEC3KT and confirmed significantly decreased PPARγ, which was associated with significantly decreased FZD9 expression (**Figure 7E**). We identified putative PPRE binding sites in the Fzd9 promoter and hypothesized that, in addition to being a target of FZD9 signaling, PAPRγ could act as a transcription factor for FZD9 (**Figure 7F**) (25). To determine if PPARγ binds to the FZD9 promoter and is sufficient to activate transcription, we transfected a PPARγ plasmid into HBEC3KT cells along with a luciferase containing the complete Fzd9 promoter and found that luciferase activity significantly increased with PPARγ compared to a control plasmid (**Figure 7G**). Transfection of PPARγ siRNA with the FZD9 promoter luciferase did not significantly decrease activity of the luciferase (data not shown), suggesting the presence of additional factors that may compensate for PPARγ loss. **Figure 7H** depicts the effects of carcinogen exposure and Fzd9 loss in lung epithelial cells. Together, these data demonstrate that PPARγ activates transcription of FZD9 and that cigarette smoke exposure may reduce FZD9 expression in part by decreasing PPARγ expression and activity.

## Discussion

Frizzled receptors have oncogenic and tumor suppressive activity depending on the specific Frizzled, tissue, or disease investigated (26). For example, FZD1 is oncogenic in lymphoma and neuroblastoma but in prostate cancer is methylated early in lesion progression, suggesting a suppressive role (27–29). In mesenchymal glioblastoma, FZD6 is overexpressed, but in gastric cancer, overexpression of FZD6 reduced tumorigenesis (30, 31). Interest in the role of FZD9 in the lung was initiated by the discovery that FZD9 and Wnt7a induced non-canonical and tumor suppressive signaling in NSCLC cells, in contrast with studies in osteosarcoma and hepatocellular carcinoma (3, 7, 32). We engineered loss of FZD9 in two HBEC cell lines and found changes in EMT genes that had previously been associated with FZD9 negative NSCLC cell lines, suggesting that persistence of early changes may contribute to lesion progression (9). EMT associated with Frizzleds may also be targeted for chemoprevention (33). We also discovered changes in the expression of new targets of FZD9, including EZH2, IL1β, and VEGFA, all of which are associated with lung cancer. EZH2 is associated with carcinogen-induced transformation of HBEC and its depletion prevents progression from hyperplasia to adenoma in an NNK mouse model (34). IL1β induces EMT in NSCLC cells and is reduced with iloprost chemoprevention (35, 36). VEGF is overexpressed in bronchial dysplasia and is associated with persistent lesions (21, 37). Increased expression of these genes in normal epithelial cells likely contributes to initiation or promotion of early lesions with FZD9 loss.

With the current study, we present the first investigation of effects of FZD9 loss in the urethane lung adenocarcinoma model. Loss of FZD9 increased tumor multiplicity *in vivo* with carcinogen exposure, however, this trend was not significant (p=0.08), which reduces the impact of this data. When the FZD9^-/-^ urethane group in this study is compared to a larger wild type urethane group from a different urethane FZD9^-/-^ study conducted in parallel (under review), there is a significant difference between adenoma number, suggesting that a larger wildtype urethane group in this study would have led to significance. Unfortunately, additional animals could not be included for this report and we acknowledge this limitation. FZD9 loss induced expression of EMT genes VIM and FN1 and inflammatory genes COX2 and IL1β. This correlates with *in vitro* data from this study and supports previous data from carcinogen exposure *in vitro* and *in vivo* (24). FZD9^-/-^ female mice exhibited higher mRNA levels of ESR1 and ESR2 in both whole lung and adenomas when compared to all male mouse groups and to wild-type female groups. This is the first observation of an association between FZD9 and estrogen in the lung. Estradiol is elevated in NSCLC, and tobacco smoke activates metabolism of 17β-estradiol to the carcinogenic metabolite 4-OH-E (19). The role of estrogen receptors in lung cancer is not as clear, but lung cancer is considered estrogen positive, with predominant ESR2 expression, and in females ESR2 is associated with a worse prognosis (38). Estrogen receptors regulate components of the tumorigenic β-catenin signaling pathway to modulate NSCLC progression (39). Increased tumor promoting characteristics in FZD9^-/-^ lung tissue and adenomas could be due to loss of regulation of estrogen receptor expression and subsequent activation of receptor signaling networks that promote cancer development. These results support investigations into the role of estrogen in development of premalignant lung lesions and the potential for anti-estrogen therapy to block progression to lung cancer.

When FZD9 binds to Wnt7a in the lung epithelium and initiates anti-cancer signaling through PPARγ, it does not activate canonical β-catenin signaling (3). In whole lung analysis from mice in this study, active β-catenin was increased with urethane but was not higher in FZD9^-/-^ tissue compared to wild type, suggesting that loss of FZD9 alone in the uninvolved tissue surrounding adenomas does not contribute to increased β-catenin signaling. In this first examination of a FZD9 negative, urethane-induced, lung adenoma cell line, we found that compared to a wildtype urethane-induced adenoma cell line, loss of FZD9 led to increased expression of apoptosis inhibiting, proliferation promoting, and EMT genes. We also found changes in protein expression that would lead to suppression of apoptosis in FZD9^-/-^ adenomas. In contrast to whole lung analysis, the FZD9^-/-^ adenoma cell line had increased active β-catenin compared to the wild type adenoma cell line, which may lead to the increased cyclinD1 observed in FZD9^-/-^ adenomas. Increased β-catenin could also lead to transcription of c-Myc, a frequently dysregulated oncogene in lung cancer (40). FZD9^-/-^ adenoma cells had increased ability to grow in an anchorage independent environment and to migrate, suggesting a more dedifferentiated phenotype. FZD9 may have a role in the development of early lung lesions and loss of FZD9 could lead to early lesions more likely to progress to carcinoma through several associated mechanisms.

In an *in vitro* model using HBEC, FZD9 expression decreases during short- and long-term CSC exposure. In cells treated with CSC followed by removal of CSC with 4 weeks of iloprost, a chemopreventive prostacyclin analogue, FZD9 expression increases while cells with continued CSC maintain low FZD9 (10). This result mirrors the oral iloprost chemoprevention trial, in which current smokers did not have improved endobronchial histology with iloprost but former smokers did (16). Examination of cell, animal, and human samples in the current study confirms that cigarette smoke exposure decreases FZD9 expression. This establishes a contribution by FZD9 to early changes in the lung epithelium that occur with cigarette smoke exposure. FZD9 also plays a key role in the activity of iloprost, where it is required for iloprost’s activation of PPARγ and downstream anti-cancer signaling (9). We demonstrated decreased activation of PPRE with FZD9 loss or CSC in HBEC and with FZD9 loss in mouse serum. Regulation of FZD9 by transcription factors in the lung is largely unknown, but Frizzleds can be regulated by non-coding RNA and we previously demonstrated that miR-31 indirectly and miR-520a-5p directly decrease FZD9 expression (10, 41, 42). Here we show evidence of PPARγ acting as a transcription factor for FZD9. FZD9 and PPARγ expression is increased by iloprost, suggesting feedback loops that propagate a response to iloprost. In the presence of cigarette smoke, however, increased FZD9 and PPARγ expression and activation are inhibited, preventing a response to iloprost and leading to persistence of early lesions in the lung epithelium. Additional studies are needed to offer a more detailed appraisal of the relationship between FZD9, PPARγ, and cigarette smoke.

Loss of FZD9 from cigarette smoke carcinogen exposure interferes with maintenance of a normal lung epithelium and could promote premalignant lesion development and progression. Recent work suggests that, contrary to previously held opinions, FZD receptors could be direct targets for small molecule drugs (43). The smoothened agonist SAG1.3 binds Fzd6 to stimulate activation and interactions in the Fzd6 signaling pathway (44). There is also evidence suggesting that the chemoprevention drug iloprost binds to FZD9 (9). FZD9 may be a target for intercepting early lung lesion development and progression. To explore the potential of FZD9 as a therapeutic or preventive target, further investigation is required to understand the regulation of FZD9 by PPARγ and other factors at the transcriptional and translational levels, and to clarify how cigarette smoke alters FZD9 expression in the lung.

## Data Availability Statement

The raw data supporting the conclusions of this article will be made available by the authors, without undue reservation.

## Ethics Statement

Ethical review and approval was not required for the study on human participants in accordance with the local legislation and institutional requirements. Written informed consent for participation was not required for this study in accordance with the national legislation and the institutional requirements. The animal study was reviewed and approved by Institutional Animal Care and Use Committee, Rocky Mountain Regional Veterans Affairs Medical Center Veterinary Medical Unit.

## Author Contributions

Conceptualization: MT, LD-N, and RK; Methodology: KK and LV; Investigation: KS, AS, AE, and L-DN; Analysis: LV, KS, AE, and MT; Funding acquisition: MT; Project administration: KS, AE, and MT; Supervision: MT and LN; Writing – original draft: KS and MT; Writing – review and editing: KS, AE, MT, L-DN, and RK. All authors contributed to the article and approved the submitted version.

## Funding

This work was supported by the National Cancer Institute (R01CA214531) (MT), an NIH CURE Diversity Supplement (AS), the Regional Mouse Genetics Core Facility (National Jewish Health and the University of Colorado Anschutz Medical Campus), and the University of Colorado Cancer Center Cell Technologies shared resource funded by the National Cancer Institute through a Cancer Center Support Grant (P30CA046934).

## Conflict of Interest

The authors declare that the research was conducted in the absence of any commercial or financial relationships that could be construed as a potential conflict of interest.

## Publisher’s Note

All claims expressed in this article are solely those of the authors and do not necessarily represent those of their affiliated organizations, or those of the publisher, the editors and the reviewers. Any product that may be evaluated in this article, or claim that may be made by its manufacturer, is not guaranteed or endorsed by the publisher.
