# Supplementary material for: Loss of Frizzled 9 in Lung Cells Alters Epithelial Phenotype and Promotes Premalignant Lesion Development
Source: Front Oncol. 2022 Jul 18;12:815737. doi: 10.3389/fonc.2022.815737 (PMC9343062; doi:10.3389/fonc.2022.815737)
Supplement: Supplementary file 1 [file DataSheet_1.docx]

Supplementary methods:

Genotyping: FZD_9_ knockout mice were developed by the Regional Mouse Genetics Core Facility at National Jewish Health and the University of Colorado. Guide design was done using CRISPR and the Broad Institute sgRNA Design software, both of which have been refined to better identify off target events. Results from each software were compared, and the guide which performed best using both algorithms was chosen. Guide activity was verified by incubating guide RNA and Cas9 protein with a PCR product containing the target sequence and comparing the ratio of cut to uncut PCR product. Zygotes were injected with guide RNA(s), Cas9, and DNA template if appropriate. Zygotes were then transferred into pseudopregnant recipients. F0 pups were genotyped by PCR using primers outside the region to be modified to identify putative positive founders. These mice were then bred to FVB/N wildtype mice. Once germline transmission was established, F1 mice were sequenced to confirm the modification and intercrossed to generate homozygous knockout mice. Fzd9-/- mice were transferred to the Rocky Mountain Regional Veterans Affairs Medical Veterinary Care Unit (RMRVAMC VMU) and animals for experiments were generated by breeding Fzd9-/- males with Fzd9-/-females to generate 100% knockout litters.

To confirm continued knock out of FZD9 in mice used for experiments, genomic DNA was extracted from ear clips of each mouse with a DNeasy Blood & Tissue kit (Qiagen). The DNA was amplified by RT-PCR with forward and reverse DNA primers and GoTaq Green Master mix (Promega) on a CFX96 Touch (Biorad). To determine FZD9 genotype, a knockout gel and wildtype gel were run. The same forward primer was used for both reactions (5’-TGCACATACAGATAGACAAGC-3’), while the knockout reverse primer (5’-GCCAGCCCGGACCTTATTTG-3’) identified the FZD9-/- sequence and the wildtype reverse primer (5’-CACTCACTGTAGCTGTCTTCAG-3’) identified WT FZD9. PCR product was analyzed by electrophoresis in precast 2.2% agarose gels at 275V (Lonza). Gels were imaged with the FlashGel™ Dock and the FlashGel™ camera using the FlashGel™ Camera and Capture software.

Western blotting: Protein was extracted using Pierce RIPA buffer (Thermo Scientific) and protease inhibitor (Thermo Scientific). The samples were quantified by BCA assay (Thermo Scientific). 20ug of protein was mixed with 2X laemmeli buffer and BME for a 1:1 sample, denatured at 56 ºC for five minutes and electrophoresed and transferred with a molecular marker according to the manufacturer’s protocol (Mini-Protean Tetra Cell, BioRad). Membranes were stained with 0.1% Ponceau S to confirm sufficient protein transfer. The membrane was blocked in 10mL of 5% Non-Fat dry milk in 1X TBS-T for 1 hour, followed by three 15-minute washes with 1X TBS-T. Blots were incubated in primary antibody (vimentin, 1:1000 Cell Signaling Technologies D21H3; COX2, 1:500 Protein Tech 6635-1-Ig; e-cadherin, 1:3000 Protein Tech 20874-1-AP) overnight at 4ºC. Membranes washed and incubated in diluted 1:5000 secondary anti-rabbit IgG antibody (BioRad) and 1:5000 StrepTactin HRP conjugate (Biorad) for 1 hour. Membranes were washed and exposed to 2mL of Clarity Western ECL substrate (Biorad) for five minutes. Images were captured and quantified with a ChemiDoc imager (BioRad). To confirm protein loading, the membrane was stripped, rinsed, blocked, and probed for β-actin (Biorad #MCA5775GA). Quantitative analysis was performed by creating a ratio between the band intensity for the protein of interest and the sample’s corresponding β-actin band intensity.

Immunohistochemistry: 5μm lung sections from urethane, iloprost, and saline groups were deparaffinized, blocked with 0.3% hydrogen peroxide, and antigen retrieval performed in boiling Diva Decloaker (Biocare Medical) under pressure for 5 minutes. Sections were blocked with Background Punisher (Biocare Medical) and 2.5% Normal Horse serum (Vector Labs). Sections were incubated in Ki67 primary Ab (1:2000 dilution, Abcam 15580) for 1 hour at room temperature, followed by anti-rabbit universal antibody for 30 minutes and ABC reagent for 30 minutes at room temperature (Vector Laboratories). Ki67+ nuclei were detected using Betazoid DAB chromagen kit (Vector Laboratories). Tumor area was measured and Ki67-positive nuclei/mm^2^ were counted in each tumor. Replicate blinded counts were conducted. The Ki67+ nuclei/mm^2^ tumor area for each tumor was averaged by group. H&E stains were done on 5μm lung sections from WT and FZD9-/- urethane and saline groups. Slides were scanned for whole lung images. Tumors were imaged on Olympus BX41 microscope fitted with an AmScope digital camera.

microCT Imaging: The FZD9-/- urethane mouse was imaged immediately before harvest. The mouse was anesthetized with isoflurane and imaged in Bruker 1276 Skyscan microCT by placing mice prone in a heated mouse bed with breathing cone and supplied with oxygen and isoflurane to maintain anesthesia. Image parameters were as follows: X-ray tube voltage of 70 kV, current of 200 μA, pixel size of 40.79 μm, exposure time of 167 ms with 0.5 mm aluminum filter and 0.7 rotation stepping. Using the computer program CTan by Bruker, the CT scan was turned into a binary image. The threshold for what tissue density was considered tumor and vessel, which are approximately the same density, vs what was considered aerated lung tissue was chosen by the person performing the data analysis. This threshold was chosen based on closely matching the binary image of the scan to the images from the actual scan. The same threshold was then applied to each scan. With CTan, an automatic series of computer operations separates the aerated lung tissue from the rest of the mouse. The lungs are then selected as a region of interest and the tumors and vessels are selected from this lung region. A 3D reconstruction was then made of the vessels and tumors and tumors were identified manually and differentiated from vessels. Vessels were removed through a combination of manual deletion and automatic computer operations.  The 3D models were then generated in CTan and visualized in the program CTvol, also by Bruker.

Supplementary Figures


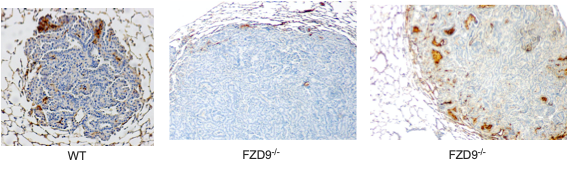


**Supplementary Figure 1**. Representative images from Ki-67 staining of adenomas from WT urethane and FZD9^-/-^ urethane mice.


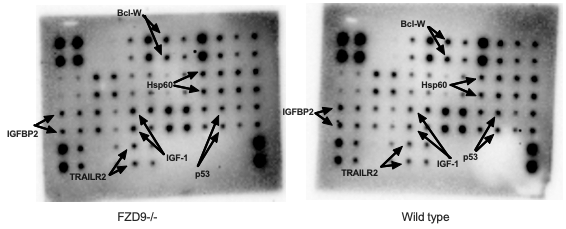


**Supplementary Figure 2**. Images of protein dot blots used to measure apoptosis signaling in wild type and FZD9^-/-^ adenoma cell lines. Targets on the graph in Fig. 5B are identified with arrows on the blots.

**Supplementary Figure 3.** Representative images from migration assay conducted with wild type (WT) adenoma and a FZD9^-/-^ adenoma cell lines. The 0hr space was created by growing cells around a 500μm silicone insert to 90% confluency and cells were imaged at 0, 4, 8, and 24 hours.
